# Comprehensive analysis of lncRNA expression profiles in cytopathic biotype BVDV-infected MDBK cells provides an insight into biological contexts of host–BVDV interactions

**DOI:** 10.1080/21505594.2020.1857572

**Published:** 2020-12-29

**Authors:** Xuwen Gao, Chao Niu, Zhuo Wang, Shuo Jia, Meijing Han, Yingying Ma, Xueting Guan, Li Wang, Xinyuan Qiao, Yigang Xu

**Affiliations:** a Heilongjiang Key Laboratory for Animal Disease Control and Pharmaceutical Development, College of Veterinary Medicine, Northeast Agricultural University, Harbin, P.R. China; b College of Veterinary Medicine, China Agricultural University, Beijing, P.R. China; c College of Animal Science and Technology & College of Veterinary Medicine, Zhejiang Agricultural and Forestry University, Hangzhou, P.R. China

**Keywords:** CP biotype BVDV, long non-coding RNAs (lncRNAs), co-expression networks, functional enrichment, signaling pathway

## Abstract

Bovine viral diarrhea virus (BVDV) is the causative agent of bovine viral diarrhea-mucosal disease, which significantly affects the production performance of cattle, causing serious economic losses to the cattle industries worldwide. Up to now, some mechanisms involved in host–BVDV interaction are still not fully understood. The discovery of long non-coding RNAs (lncRNAs) has provided a new perspective on gene regulation in diverse biological contexts, particularly in viral infection and host immune responses. However, little is known about the profiles and functions of lncRNAs in host cells in response to BVDV infection. Here, we utilized Illumina sequencing to explore lncRNAs profiles in cytopathic (CP) biotype BVDV-infected MDBK cells to further reveal the potential roles of lncRNAs in BVDV infection and host–BVDV interaction with integrated analysis of lncRNAs and mRNA expression profiles. A total of 1747 significantly differentially expressed genes, DEGs (156 lncRNAs and 1591 mRNAs) were obtained via RNA-seq in BVDV-infected MDBK cells compared to mock-infected cells. Next, these DE lncRNAs and mRNAs were subjected to construct lncRNAs-mRNAs co-expression network followed by the prediction of potential functions of the DE lncRNAs. Co-expression network analysis elucidated that DE lncRNAs were significant enrichment in NOD-like receptor, TNF, NF-ĸB, ErbB, Ras, apoptosis, and fatty acid biosynthesis pathways, indicating that DE lncRNAs play important roles in host–BVDV interactions. Our data give an overview of changes in transcriptome and potential roles of lncRNAs, providing molecular biology basis for further exploring the mechanisms of host–BVDV interaction.

## Introduction

Bovine viral diarrhea-mucosal disease (BVD-MD) caused by bovine viral diarrhea virus (BVDV) is one of the most important bovine infectious diseases and is found in many cattle-producing countries worldwide. BVDV is an enveloped single-stranded positive-sense RNA virus with a genome of approximately 12.3 to 12.5 kb, similar to classical swine fever virus and border disease virus, belonging to the genus *Pestivirus*, family *Flaviviridae* [1,2]. BVDV infection seriously affects respiratory system and digestive system of cattle, thereby compromising the production performance of the herd. The most serious hazard of this disease is the formation of persistent infection (PI) in fetal cattle infected with BVDV during pregnancy, which can cause immune tolerance in cattle [3]. Moreover, PI cattle continuously excrete large amounts of viruses throughout their life process, causing BVDV to spread among the herd for a long time, making it extremely difficult to eliminate, which has resulted in large economic losses to the cattle industry worldwide [4].

According to the cytopathic effect (CPE) of BVDV-infected cells, BVDV is divided into two biotypes, cytopathic (CP) biotype, and non-cytopathic (NCP) biotype [5]. Among them, the CP or NCP biotype BVDV strains are divided into BVDV-1, BVDV-2, and atypical BVDV genotypes based on the viral sequence variation with an amino acid homology of approximately 85% [6–8]. Epidemiological investigations have showed that these BVDV biotypes and genotypes are widespread in countries and regions with developed cattle husbandry [9,10]. In North American, the positive rate of BVDV infection is approximately 9.72–26.53%, while in Europe, the rate is 40.82–53.47% [11]. China has one of the highest BVDV infection rates, with over 46.7% of cattle farms testing positive for the presence of BVDV with a PI rate of approximately 2.2% in the herds [12]. Recently, an analysis for the epidemiological investigation data of BVDV infection from the major cattle-rearing provinces in China showed that the overall BVDV positive rate was 58.09%, testing for the presence of BVDV-specific antibodies using ELISA [13]. These findings indicate the serious hazard posed by BVDV to the development of the cattle industry in China.

In addition to cattle, BVDV can also infect sheep, pigs, deer, and other wild animals [14–16]. Although there is currently no effective cure for the disease caused by CP biotype BVDV infection in animals, existing BVDV vaccines contribute to effectively preventing the disease epidemic [17]. However, vaccinated and naturally infected animals cannot be effectively distinguished by serological methods. The identification and elimination of PI livestock to prevent the spread of BVDV is the most widely used strategy today. However, this inevitably results in important economic losses for the farms due to the need to detect and remove PI animals, and underdeveloped countries and regions cannot bear such heavy costs. Thus, the elucidation of the mechanism of BVDV infection and host immune responses is crucial to characterize the pathogenesis of BVDV and to develop vaccines with which to control BVDV infection.

Long non-coding RNAs (lncRNAs) are a class of non-coding RNA with a length of over 200 nucleotides (nt). They are located in the cytoplasm or nucleus and play an important role in biological activities and disease development by regulating target genes, thereby affecting gene expression [18,19]. Although a recent study reported that certain lncRNAs are able to encode small peptides [20], lncRNAs generally lack an efficient open reading frame, such that they do not perform a protein-coding function. LncRNAs tend to fold into thermodynamically stable secondary or higher-order structures that provide multiple sites within the molecule for the binding of proteins. Specific and dynamic interactions can also occur between DNA and RNA through the principle of base-complementary pairing. Some lncRNAs could be cleaved into polyA tails and promoter structures similar to mRNA and are dynamically expressed during tissue organ differentiation [21] followed by formation of a complex and precise gene expression regulatory networks. Many studies have reported the functional activities of lncRNAs in the innate immune system [22,23], adaptive immunity [24], the tumor development process [25,26], and in several human diseases [27]. Current research indicates that lncRNAs are a key regulator of viral infection and host immune responses involved in host–virus interaction [28,29]. However, the profiles and functional activities of lncRNAs in host cells in response to BVDV infection have not yet been elucidated.

Here, Illumina sequencing was employed to explore the different expression profiles of lncRNAs in BVDV-infected MDBK cells for further investigating the roles of lncRNAs in BVDV infection and host–BVDV interaction. The potential functions of the differentially expressed lncRNAs were predicted by lncRNA-mRNA co-expression network, which would provide a molecular biology basis for further understanding the roles of lncRNAs in host–BVDV interaction.

## Materials and methods

### Cell and virus

Bovine kidney (MDBK) cells were cultured with high-glucose medium (Hyclone, USA) supplemented with 10% fetal bovine serum (FBS) (Gibco, USA) at 37°C in a 5% CO_2_ incubator. CP biotype BVDV strain VEDEVAC AV69 kept in our laboratory was propagated on MDBK cells, and the virus titer was approximately 10^6.5^ 50% tissue culture infectious dose, TCID_50_ per 0.1 mL. Before infecting the MDBK cells with BVDV, 2 × 10^5^ cells/mL of MDBK cells were seeded into 6-well plates and cultured at 37°C in a 5% CO_2_ incubator for 24 h. Subsequently, 10^3^ TCID_50_ BVDV was added to each well (experimental group, E group). In parallel, high-glucose medium was used for mock infection (control group, C group).

### RNA extraction and library construction

**Figure 1.**
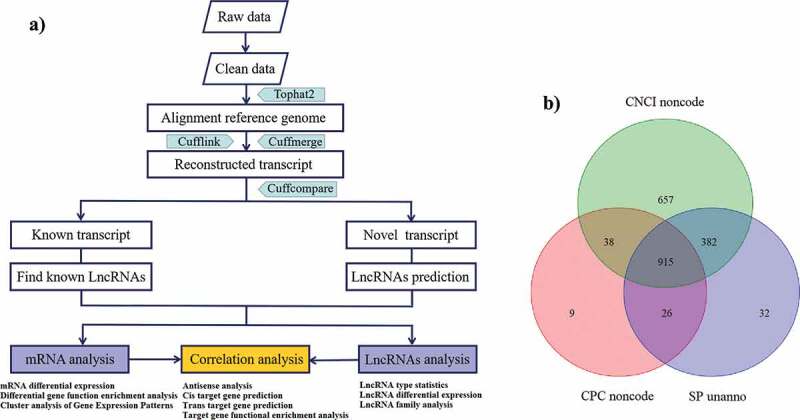
Overview of RNA sequencing. (a) The workflow of identification pipelines for lncRNAs. (b) New transcripts were predicted using CPC and CNCI software. These transcripts were compared to the protein database SwissProt

After infecting with BVDV for 48 h, with three parallel samples in each group, the MDBK cells were collected from the E and C groups, followed by total RNA extraction by TRIzol (Invitrogen, USA), according to the manufacturer’s instructions. The purity, concentration, and integrity of the total RNA was evaluated using Agilent Bioanalyzer 2100 (Agilent Technologies, Santa Clara, CA, USA). The samples with RNA integrity number (RIN) value of >9.7 and optical density 260:280 ratio of >2.0 were selected for library construction and deep sequencing. For the construction of lncRNA libraries, ribosomal RNAs (rRNAs) were removed from the total RNA extractions using Ribo-zero TM rRNA Removal Kit (Epicentre, Madison, WI, USA). The enriched messenger RNAs (mRNAs) and ncRNAs were fragmented into cDNA using random primers (Thermo Fisher Scientific, USA). With the synthetization of second-strand cDNA, the cDNA fragments were purified by QiaQuick PCR extraction kit (Qiagen, Germany) before adding the end-repaired poly (A), and then ligated to Illumina sequencing adapters. Subsequently, the second-strand cDNAs were digested by uracil-N-glycosylase, and the digested products were size-selected using agarose gel electrophoresis followed by PCR amplification. Gene sequencing was performed using Illumina HiSeq^TM^ 4000 and bioinformatics analysis was supported by Gene Denovo Biotechnology Co., Ltd. (Guangzhou, China). The protocol for the transcript analysis is shown in Figure 1(a). Briefly, after filtering the off-machine raw data, clean reads were obtained followed by alignment to the reference genome; Next, use Cufflinks to reconstruct transcripts to obtain the known transcripts and the new transcripts; Next, use CPC, CNCI and other software to predict the coding ability of the new transcripts to obtain the newly predicted lncRNAs; And then, perform analysis of the mRNAs and lncRNAs, respectively; Finally, perform the association analysis of lncRNAs-mRNAs. All raw sequence reads were deposited into the NCBI database as a BioProject (accession no. PRJNA596327).

### Target gene prediction and functional analysis

RNAhybrid (v2.1.2) + svm_light (v6.01), Miranda (v3.3a), and TargetScan (v7.0) software were used to predict target genes. Next, the predicted genes were submitted to Gene Ontology (GO) database analysis for functional annotation analysis, and Kyoto Encyclopedia of Genes and Genomes (KEGG) database analysis to identify the enriched pathways involved. And, fisher’s exact test and chi-square test were used to determine the significance of the GO terms and pathways. The false discovery rate (FDR) was calculated to correct the P-value, and only the GO terms and pathways with an adjusted P-value of ˂0.05 and an FDR <0.05 were selected.

### LncRNA-mRNA association analysis

LncRNAs are involved in many post-transcriptional regulation processes, where some anti-sense lncRNAs may regulate gene silencing, transcription, and mRNA stability. In order to reveal the interactions between antisense lncRNAs and mRNAs, RNAplex software (http://www.tbi.univie.ac.at/RNA/RNAplex.1.html) was used to predict the complementary correlation of antisense lncRNAs and mRNAs. One of functions of lncRNAs is the cis-regulation of neighboring genes on the same allele. The up-stream lncRNAs that intersect with the promoter or other cis-elements may regulate gene expression at the transcriptional or post-transcriptional level. The downstream or 3ʹUTR region lncRNAs may have other regulatory functions. For lncRNA cis-regulation analysis, the lncRNAs that had been annotated previously as “unknown region” were annotated again. LncRNAs found less than 100 kb up- or down-stream of a gene were likely to be cis-regulators. The cis target genes were then subjected to enrichment analysis of GO functions and KEGG pathways. For lncRNA trans-regulation of co-expressed gene analysis, which is another function of lncRNAs, the correlation of expression between lncRNAs and protein-coding genes was analyzed to identify the target genes of lncRNAs. Pearson correlation coefficient was used for samples ≥6 and protein-coding genes with absolute correlation >0.9 were then subjected to enrichment analysis of GO functions and KEGG pathways. The enrichment analysis of the GO functions and KEGG pathways were then conducted in protein-coding genes in the network.

### Validation of selected lncRNAs and mRNAs by qPCR

To validate the accuracy of the Illumina sequencing data, thirteen DE mRNAs, and eight DE lncRNAs were selected at random and examined by RT-qPCR assay with SYBR Green Master (Takara, Dalian, China) on a 7500 real-time PCR system (ABI, USA). PCR primers were designed according to the target mRNAs and lncRNAs using Oligo 7.0 software, whose specificity was evaluated by BLAST analysis and smelting curve analysis of amplicons, and only the primers can be used until amplification efficiency reached 90–110%. The details of the specific primers used in this study are listed in Table 1. The β-actin was used as an internal reference gene to normalize the expression of the selected target mRNAs and lncRNAs. All qPCRs were performed in three technical replicates.

**Table 1.** RT-qPCR primers used for the verification of mRNAs and lncRNAs

mRNAs		LncRNAs	
Gene	Primer sequence	Gene	Primer sequence
β-ACTIN	F: GCCAACCGTGAGAAGATGAC R:AGGCATACAGGGACAGCACA	β-ACTIN	F:GCCAACCGTGAGAAGATGAC R:AGGCATACAGGGACAGCACA
BIRC2	F:GTGTTGAGACAAGGTCCTGGTTCC R:TGATTGGTTACTGGTGAGGCAACG	LOC104969159	F:TCTCGCTGCATCTCTAACGG R:CCATGGTCAGGACTGTCTCCA
IGF1R	F:AACATCGCTTCGGAACTGGAGAAC R:TCAGGAAGGACAAGGAGACCAAGG	LOC101905498	F:CAACAAGGAATGCCAACGG R:GGAGGCCAGAAGTCCAAAACA
PTCD2	F:TCTGGTTCTACCTGCTGCCTCTG R:ACATGCAACAGCCACCTTCTTACG	LOC112444644	F:GCTGTTCTCCTCCTTCTATCTCC R:GAGGGTGTCAGAGGGTCCAA
DTX2	F:AGCGTCTGCGACTACCTGGAG R:AAGTCTTGTTGGTCTGCGTGTGG	LOC101902030	F:CCCTGCCCTACGATTCTTG R:TCCGTGGTTTGGAGTTGCTAT
GTF2I	F:GAGCAGGATGGTGGTGACGTTC R:GCACGCTACTTCCGCCTTGG	LOC101906383	F:AGGCTCACTGATTGTGGCTAA R:GAAGATGTGGTCCACGAAGG
MAPK8	F:ATTGAGCAGAGCAGGCATAAGTGG R:CCGTCAGGAGCAGCACCATTC	LOC104974530	F:ACTTACTGGCATAAAGACACC R:AACCTCCTAACCATCACATC
FGFR1	F:CACCAACCTCTAACCGCAGAACTG R:GTCATCGTCGTCATCATCGTCCTC	TCONS00071017	F:CGCTATCGGAGTTCACGG R:TGGAATACAGGGTTTCACAGG
IKBKG	F:GAGCAGCATGGTGAGCAGTGAG R:TGTTCCGCCTCCTCCTTCAGC	TCONS00026615	F:CGGTAGTTAAACAGCACCTGAC R:TGGGCTTATCACTGGGAGAC
RPS6KA2	F:CGTGTAGGTGTGAGTGCTGAGTG R:CACAACTGTCCGCTGTCTCCTG	LOC112444864	F:AAGGAACTGGGTGTTTGGAGA R:TGGAGCACGGGCTGAGTA
MAP3K4	F:ACTAAGTGTGAGAGCGGCAGAGG R:GCAGAGCAGAGATGAACGAAGGC		
CACNA1G	F:GCCTCCTGTGTCACCGAATCATC R:CGCCACCACCTTCACTGTCATC		
IL1R1	F:TTCCAAGTCACCTCCTCCTCTCAC R:GCGTGTGCAGAGCAGGACTG		

## Statistical analysis

Data for qPCR are presented as mean ± standard error of three replicates per test in a single experiment repeated three times. Comparisons between the two groups were performed using the Student’s t-test. Comparisons of multiple group data were performed using one-way analysis of variance (ANOVA) followed by Tukey’s post-hoc test. P-values <0.05 were considered statistically significant. Statistical values were calculated using SPSS software, version 20.0 (IBM Corp., USA).

## Results

### Overview of RNA sequencing (RNA-seq)

To identify the RNAs expressed in BVDV-infected MDBK cells, two cDNA libraries (C group: mock-infected MDBK cells group; E group: BVDV-infected MDBK cells group) were constructed. Each group was tested using three biological replicates. A highly stringent filtering pipeline was used to remove uncharacterized transcripts from the downstream analysis in RNA-seq. The workflow for the transcript analysis is shown in Figure 1(a). The final library was subjected to sequencing by Illumina HiSeq^TM^ 4000. A total of 78,886,865,100 raw reads were generated in all libraries. After discarding the adaptor sequences, ribosomal RNAs, and low-quality reads, we obtained 519,821,014 clean reads, which were aligned to the reference genome of the species using the alignment software Tophat2 (2.1.1).

### Differentially expressed mRNAs in BVDV-infected MDBK cells

**Figure 2.**
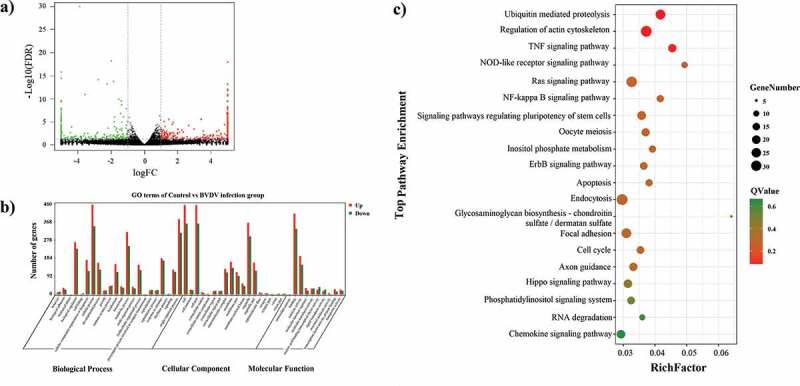
RNA-Seq analysis of mRNA in BVDV-infected MDBK cells. (a) Volcano plot map of mRNA expression in BVDV-infected MDBK cells compared to mock-infected cells. The abscissa indicates the log of the difference multiple of the two samples. The ordinate indicates the negative Log_10_ value of the FDR of the two samples; the red points (up-expression) and green points (down-regulation) indicate the difference in gene expression (judgment criteria: FDR <0.05, and the difference multiple is more than twice); there is no difference in black dots. (b) GO term classification statistics were performed on the differential genes. The abscissa represents the three ontologies of GO: molecular function, cellular component, and biological process; the ordinate represents the number of genes corresponding to each classification entry. (c) KEGG enrichment analysis between groups. “RichFactor” refers to the ratio of the number of transcripts in the pathway entry in the DE transcripts to the total number of transcripts located in the pathway entry. The larger the RichFactor, the higher the degree of enrichment. Q-value indicates the P-value after multiple hypothesis test corrections, ranging from 0 to 1; the closer to 0, the more significant the enrichment. The figure was plotted using the Q-value from small to large for the top 20 paths

Difference analysis of mRNAs expression between the E and C groups was performed using edgeR software. In order to explore the similarities and compare the relationships between the different libraries, the expression patterns of differentially expressed (DE) mRNAs were measured using systematic cluster analysis (Supplementary Figure S1a, Figure S2a, and Figure S3). According to the cutoff criteria (P < 0.05 and |log2FC|>1), a total of 895 up-regulated genes and 696 down-regulated genes were identified in the BVDV-infected MBDK cell group (Figure 2(a)). Then, Gene Ontology (GO) categorization and pathway analysis of the differentially expressed genes (DEGs) were performed using the online software package. To better understand the potential roles of host factors involved in BVDV infection, GO term classification statistics involved in molecular functions, cellular components, and biological processes was performed for the DEGs that were up-regulated or down-regulated (Figure 2(b)). Of which most significantly enriched GO terms related to biological processes were cellular component organization or biogenesis, biological regulation, metabolic process, cellular process, response to stimulus, and single-organism process; the majority of DEGs in comparison group E vs. C were associated with stimulation related to cellular component, including cell, cell part, organelle, macromolecular complex, and membrane; the majority of DEGs were assigned to molecular function associated with stimulus, including binding, catalytic activity, molecular function regulator, molecular transducer activity, and transporter activity.

**Figure 3.**
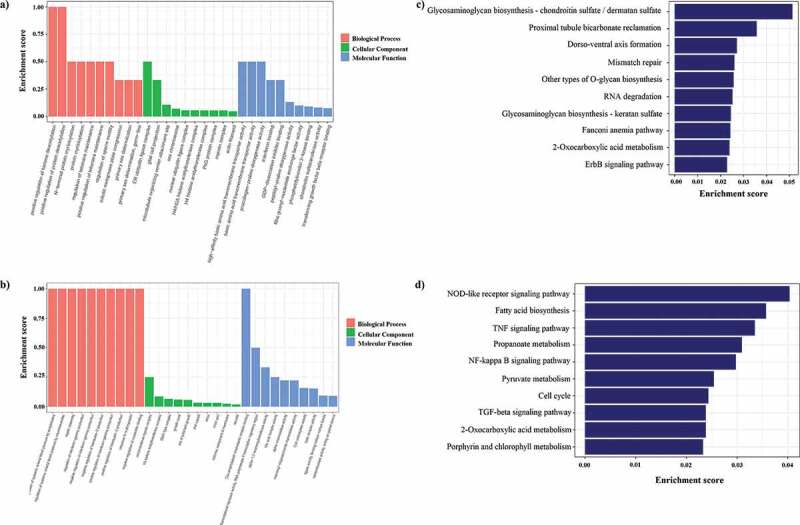
Gene Ontology (GO) and pathway analysis. (a) GO annotation of up-regulated mRNAs with top ten enrichment scores. (b) GO annotation of down-regulated mRNAs with the top ten enrichment scores. (c) KEGG pathway enrichment analysis of up-regulated mRNAs with top ten enrichment scores. d. KEGG pathway enrichment analysis of down-regulated mRNAs with top ten enrichment scores

**Figure 4.**
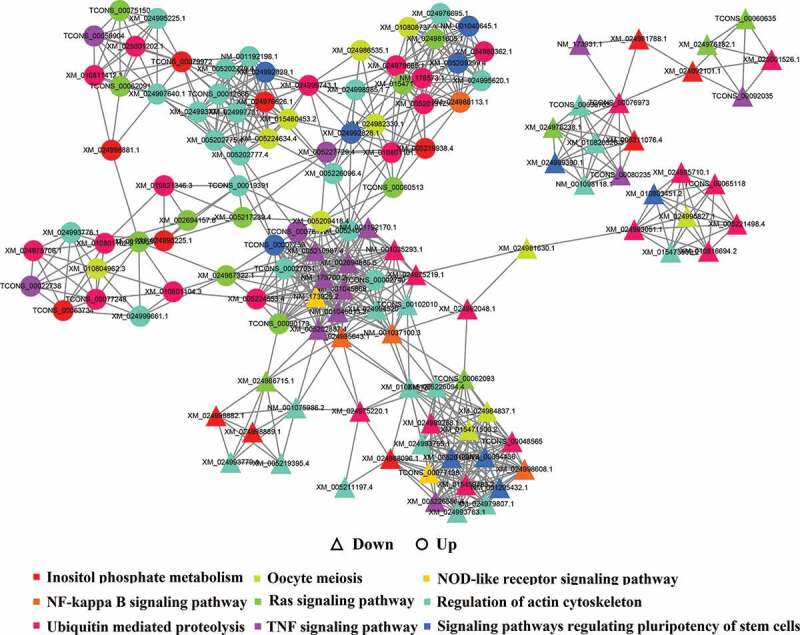
The link and overlapping of associated molecules among significant pathways (inositol phosphate metabolism, Oocyte meiosis, NOD-like receptor signaling pathway, Ras signaling pathway, Fatty acid biosynthesis, NF-kappa B signaling pathway, Regulation of actin cytoskeleton, Ubiquitin mediated proteolysis, TNF signaling pathway, etc.) with top ten enrichment scores. significant pathways

It is well known that signaling pathway analysis can help to better understand the biological functions of genes, and KEGG pathway enrichment analysis of DEGs can be used to determine the biochemical metabolic and signal transduction pathways, which further facilitate us to explore the host–virus interactions. So we also performed signaling pathway analysis using the KEGG databases, the results of which showed that the genes differentially regulated by BVDV infection were mainly involved in TNF signaling pathway, NF-κB signaling pathway, NOD-like receptor signaling pathway, apoptosis signaling pathway, ErbB signaling pathway, fatty acid biosynthesis pathway, and Ras signaling pathway, compared to those in the C group (Figure 2(c)), indicating that BVDV infection affects host immune responses. Then, the up-regulated mRNAs (Figure 3(a)) associated with the top 10 signaling pathways (Figure 3(c)) and the down-regulated mRNAs (Figure 3(b)) associated with the top 10 signaling pathways (Figure 3(d)) were analyzed. The correlation and overlap of the related molecules in the top 10 signaling pathways related to mRNA up-regulation and the top 10 signaling pathways related to mRNA down-regulation were analyzed. As shown in Figure 4, DEGs including IL1R1, PPP3CC, BIRC2, LPIN1, TIAM1, MAPK8, IGF1R, JAK1, CYLD, ACLY, HMGCR, ACSS2, and MAP3K1, etc., are mainly enriched in NF-κB signaling pathway, TNF signaling pathway, and NOD-like receptor signaling pathway, and Fatty acid biosynthesis, etc., suggesting that BVDV infection not only affects the host immune responses but also affects lipid synthesis and metabolism activity in host cells.

### Identification of lncRNAs in BVDV-infected MDBK cells by RNA-seq

**Figure 5.**
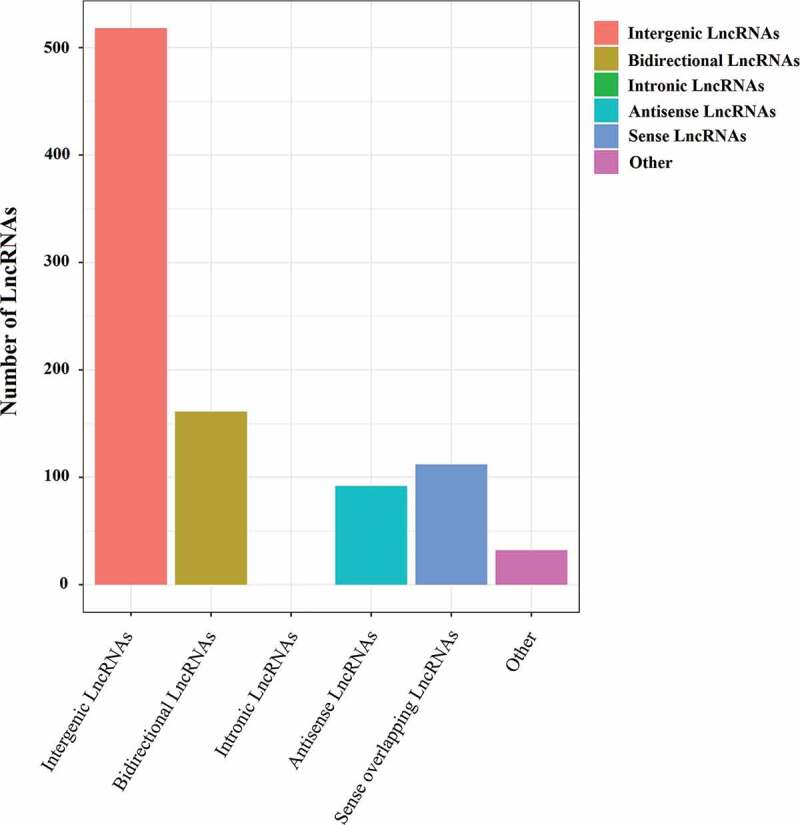
Novel types of lncRNA transcripts. According to the position of the lncRNAs on the genome relative to the protein-encoding genes, the novel lncRNAs can be divided into five categories: intergenic lncRNAs, which locate between annotated protein-coding genes and are at least 1 kb away from the nearest protein-coding genes; bidirectional lncRNAs, which are oriented head to head with a protein-coding gene within 1kb; introns lncRNAs, which overlap with the intron of annotated coding genes in either sense or antisense orientation; antisense lncRNAs, which are transcribed from the antisense strand and overlap in part with well-defined spliced sense or intronless sense RNAs; sense-overlapping lncRNAs, which are considered transcript variants of protein-coding mRNAs, as they overlap with a known annotated gene on the same genomic strand

The transcripts were reconstructed using Cufflinks based on the alignment results of Tophat2 software. Basic lncRNAs screening was performed based on the following screening criteria: transcript length ≥200 bp and exon number ≥2. The coding ability of the new transcript was predicted using CPC and CNCI software and was further compared to the protein database SwissProt. Only transcript intersections without coding potential and protein annotation information were taken as reliable prediction results. Then, new lncRNA predictions were performed from these newly selected transcripts. Our pipeline yielded 3981 lncRNA transcripts, including 3066 known isoform lncRNAs and 915 new isoform lncRNAs (Figure 1(b)). As shown in Figure 5, according to the position of new lncRNAs in the genome relative to protein-encoding gene, these new lncRNAs could be divided into five categories: intergenic lncRNAs, bidirectional lncRNAs, intron lncRNAs, antisense lncRNAs, and sense-overlapping lncRNAs.

### Identification of DE lncRNAs in BVDV-infected MDBK cells

**Figure 6.**
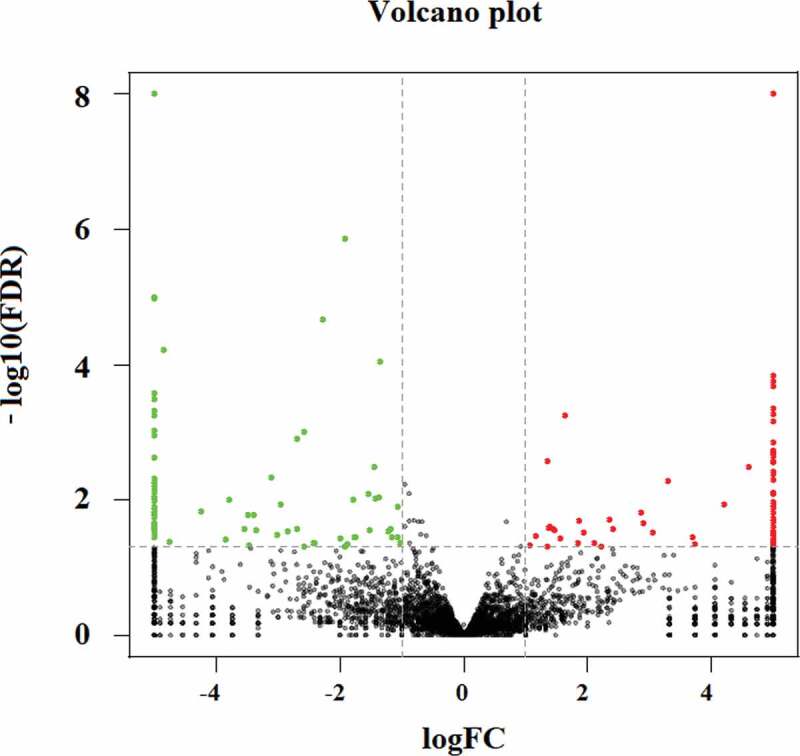
Volcano plot map of lncRNAs expression in BVDV-infected MDBK cells compared to mock-infected cells. The abscissa indicates the log of the difference multiple between two samples; the ordinate indicates the negative Log_10_ value of the FDR of two samples; the red points (up-regulation) and green points (down-regulation) indicate the differences in gene expression (judgment criteria: FDR < 0.05; the difference multiple is more than twice). Black dots indicate that there is no difference

The changes in expression profiles of the lncRNAs in BVDV-infected MDBK cells were determined. The expression levels of the lncRNA transcripts were estimated using FPKM (fragments per kilo-base of exon per million fragments mapped), which was calculated using Cufflink software. The differential expression analysis of the lncRNAs between groups was performed using edgeR software. According to the cutoff criteria (P < 0.05 and |log2FC|>1), a total of 156 significantly DE lncRNAs, including 72 up-regulated lncRNAs and 84 down-regulated lncRNAs upon stimulation with BVDV, were identified (Figure 6). To explore the similarities and compare the relationships between the different libraries, the expression patterns of the DE lncRNAs were measured using systematic cluster analysis (Supplementary Figure S1b, Figure S2b, and Figure S4).

### Analysis of the complementary binding between antisense lncRNA and mRNA

**Figure 7.**
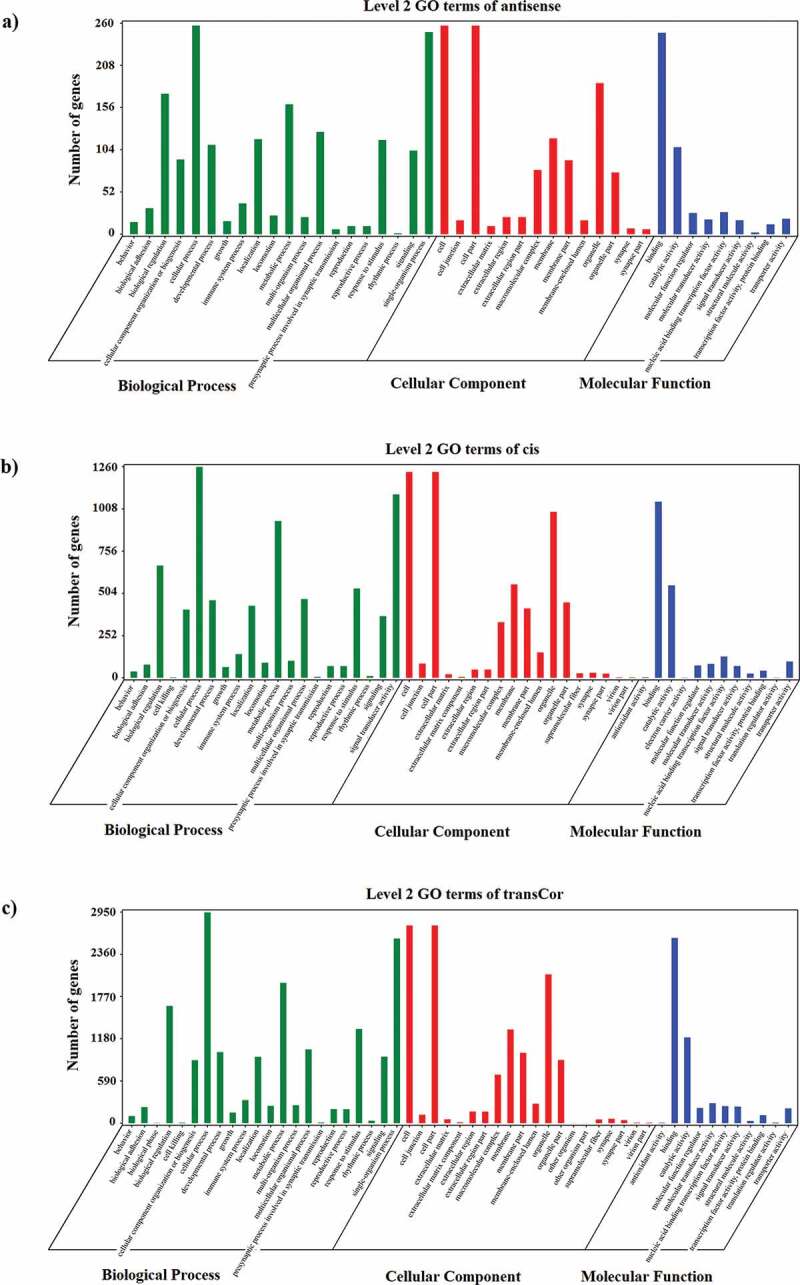
GO enrichment analysis of lncRNAs and mRNAs. (a) GO enrichment result of antisense target genes with top ten enrichment scores; (b) GO enrichment result of cis target genes with top ten enrichment scores; (c) GO enrichment result of trans-targeting genes with top ten enrichment scores

**Figure 8.**
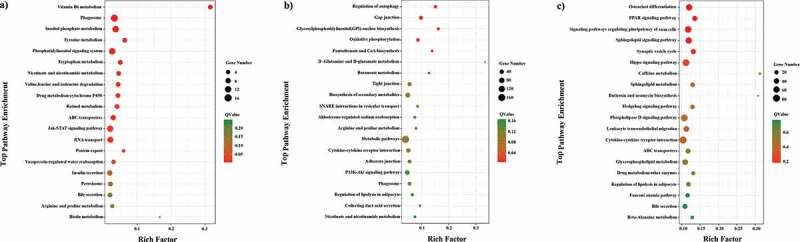
Signaling pathway enrichment analysis of lncRNAs and mRNAs. (a) Pathway enrichment of antisense target genes with top ten enrichment scores; (b) Signaling pathway enrichment of cis-targeting genes with top ten enrichment scores; (c) Pathway enrichment of trans-targeting genes with top ten enrichment scores

To reveal the interaction between antisense lncRNAs and mRNAs, RNAplex software that calculates the minimum base pairing energy based on its thermodynamic structure was used to predict the optimal base-pairing relationship for short interactions between two long-chain RNAs, as well as to predict complementary binding between antisense lncRNA and mRNA. Our data showed that there were 568 predicted interactions between 304 antisense lncRNAs and 289 mRNAs. We found that 43 GO terms were significantly enriched (P < 0.05) (Figure 7(a)) and 174 candidate antisense target genes were enriched in 110 KEGG pathways, including Phagosome, Jak-STAT signaling pathway, and RNA transport, which were included in the top 20 enriched terms (Figure 8(a)). Based on these data, we further obtained other metabolic-related genes enriched in biosynthesis of secondary metabolites, oxidative phosphorylation, glycosylphosphatidylinositol (GPI)-anchor biosynthesis and other pathways. These results indicated that one of the principal roles of lncRNAs might be the transcriptional regulation of gene expression.

### Enrichment analysis of nearest neighbor genes of the lncRNAs

To investigate possible functions of the lncRNAs, we predicted potential target genes of lncRNAs in cis-regulatory relationships. We searched for protein-coding genes 10-kb upstream and downstream of all the identified lncRNAs and found 1683 lncRNAs that were transcribed close to (<10 kb) 803 protein-coding neighbors. Using GO analysis for the cis lncRNA targets, we found 51 GO terms that were significantly enriched (P < 0.05) (Figure 7(b)). Signaling pathway analysis showed that 801 candidate cis target genes were enriched in 209 KEGG pathways, where the top 20 enriched terms included regulation of autophagy, cytokine-cytokine receptor interaction, and PI3K-Akt signaling pathway, among others (Figure 8(b)).

### Enrichment analysis of co-expressed genes of lncRNAs

**Figure 9.**
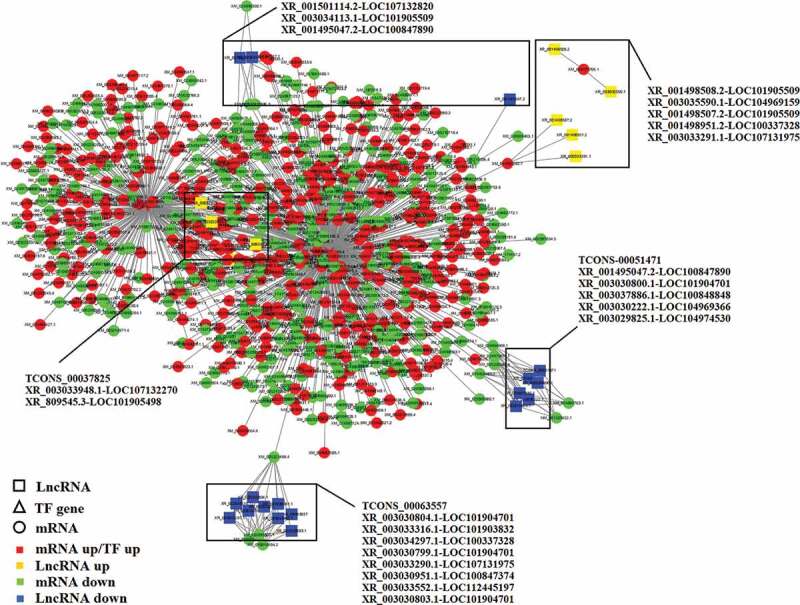
The lncRNA-TFs-mRNA regulation network

We predicted the potential targets of lncRNAs in trans-regulatory relationships using co-expression analysis. A total of 6121 interaction relationships were found, including 3260 positive correlations and 2861 negative correlations between 3981 lncRNA transcripts and 1958 protein-coding transcripts that corresponded to 23366 protein-coding genes in the *Bos taurus* reference genome. The enrichment analysis showed that the co-expressed genes were enriched in 53 GO terms, including 22 terms under biological process, 11 terms under molecular function, and 20 terms under cellular component (Figure 7(c)). Significantly, correlation-immune functions, such as MAP kinase activity (GO: 0004707), interleukin-12 binding (GO: 0019972), nuclease activity (GO: 0004518), and nucleotide transmembrane transporter activity (GO: 0015215), were included in some GO terms. In addition, the co-expressed genes were enriched in 225 KEGG pathways, including NOD-like receptor signaling pathway, T cell receptor signaling pathway, NF-κB signaling pathway, fatty acid biosynthesis, MAPK signaling pathway, and PPAR signaling pathway (Figure 8(c)), indicating that lncRNAs can regulate trans-target genes. Since lncRNAs may exert their functions by regulating co-expressed genes, we identified a group of co-expressed lncRNAs/genes based on the results of the top 10 signal pathways in previous pathway enrichment, and mapped their interactions (Figure 9).

### Validation of DE lncRNAs and mRNAs by qPCR

**Figure 10.**
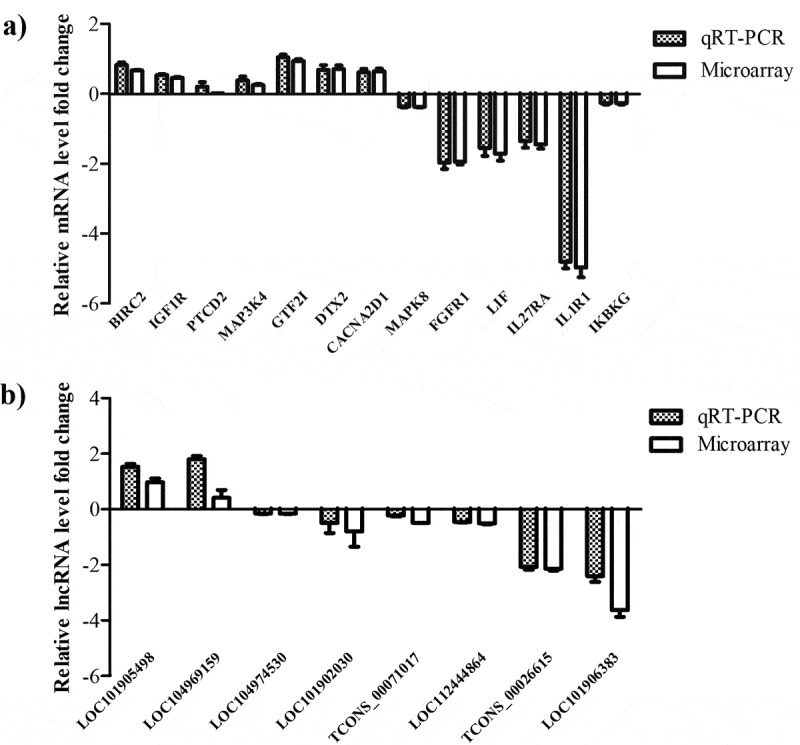
Validation of the DE mRNAs and lncRNAs by RT-qPCR. (a) Expression levels of DE mRNAs were validated by RT-qPCR. (b) Expression levels of DE lncRNAs were validated by RT-qPCR. Bars presents as mean ± standard error of three replicates per test in a single experiment repeated three times

In order to confirm the expression profiles of the mRNAs and lncRNAs in BVDV-infected MDBK cells obtained from the Illumina sequencing analysis, we randomly selected eight DE lncRNAs and thirteen DE mRNAs to verify by qPCR assay. The qPCR results confirmed the Illumina sequencing data, showing similar trends in the up- or down-regulated mRNAs (Figure 10(a)) and lncRNAs (Figure 10(b)).

## Discussion

BVDV has caused huge economic losses to the livestock industry. Although great progress has been made in the research of BVDV in recent decades and several signaling pathways closely related to BVDV infection have been reported [30–32], the infection and pathogenesis of BVDV are so extremely complex that the relevant mechanisms have yet to be comprehensively characterized. In recent years, lncRNAs have been found to play an important role in regulating viral infection and host immune responses involved in host–virus interaction. However, the profiles and functional activities of lncRNAs in host cells in response to BVDV infection have not been elucidated. In this study, we determined the changes in the expression profiles of lncRNAs and mRNAs in BVDV-infected MDBK cells. As a result, differentially expressed lncRNAs and mRNAs were identified, providing a new perspective on gene regulation in the host–BVDV interactions. Moreover, the expression changes of randomly selected DE lncRNAs and DE mRNAs were confirmed by qPCR assay, indicating the reliability of the Illumina sequencing results.

The metabolism can provide energy and materials required for all biological processes, such that the balance between catabolic and anabolic pathways is essential to meet various biological needs of living organisms [33]. Viruses are obligate intracellular parasites that obtain all energy and materials necessary for infection and replication in hosts. Generally, viruses can generate their own suitable intracellular microenvironment for their viral life cycle by altering the host’s cellular metabolic network [34,35]. By analyzing the DEGs, we found that the levels of IGF1R, ACLY, HMGCR, HMGCS1, ACSS2, INSIG1, and LPIN1 genes were significantly up-regulated during the BVDV life cycle. The IGF-1 receptor (IGF1R) binds with highest affinity to the insulin-like growth factor-1 (IGF-1), while ligand binding, in turn, regulates gene transcription, as well as the glucose, lipid, and protein metabolisms, in addition to cell growth and differentiation [36]. The ACLY gene is related to intramuscular fat deposition in cattle, which is of great significance for improving the quality of breeding beef [37]. HMGCR, HMGCS1, and ACSS2 are mainly associated with improved growth performance and meat quality [38–41]. The lipid phosphate phosphohydrolase (LPIN), also known as the lipoprotein gene, is a family of genes that can bidirectionally regulate the fat metabolism [42,43]. LPIN1 is a key gene for regulating the formation and differentiation of adipose tissue, and studies have shown that non-expression, normal expression, and over-expression of the LPIN1 gene can cause dramatic changes in fat deposition [44,45]. INSIG, SCAP, and SREBPs together constitute the INSIG-SCAP-SREBPs transfer system, which is stored in the form of complexes in the endoplasmic reticulum. Intracellular cholesterol can affect the activation of the INSIG-SCAP-SREBPs, which in turn regulates the transcription of low-density lipoprotein receptors. Ultimately, feedback regulates the cellular intake of exogenous cholesterol [46]. Significantly, these up-regulated genes are mainly related to the lipid metabolism, indicating that BVDV may alter the host lipid metabolism to promote self-survival during BVDV life cycle. Further investigation *in vivo* and *in vitro* will be required to confirm these findings.

In this study, 156 significantly DE lncRNAs in BVDV-infected MDBK cells were obtained, including 72 up-regulated lncRNAs and 84 down-regulated lncRNAs. We found that the location of the lncRNAs in the genome appeared to be nonrandom, indicating a link between lncRNA function and genomic location. Using RNA interference, a previous study found that a group of human lncRNAs act as enhancers to activate their adjacent genes, such as SCL, SNA1, and SNA2 by cis action [47], demonstrating the co-expression relationship between lncRNA and its nearby protein-coding genes. Other findings have reported that the lncRNA-mediated cis-regulation of adjacent gene transcription is ubiquitous and a major mode in gene regulation of mammalian [48]. Therefore, understanding the cis-regulation of lncRNA is crucial to reveal the biological significance of lncRNAs. In this study, several lncRNAs (LOC112443855, LOC112448807, LOC100847453, and LOC101904128, among others) were identified in cis-regulation with apoptosis-related protein-coding genes during BVDV infection. Autophagy is also a crucial mechanism in host resistance to infection, and several studies have reported that BVDV infection significantly increased cellular autophagy in MDBK cells [49,50]. Our Illumina sequencing data also demonstrated that some lncRNAs targeted autophagy-related genes ATG4D, ATG16L1, ATG16L2, and ATG5. Moreover, lncRNA provides more complexity for gene regulatory networks and some lncRNAs function through trans-acting, such as LOC107132573. We also found that the expression levels of several lncRNAs were significantly increased in BVDV-infected MDBK cells, including LOC101902030, LOC107132820, LOC107131785, LOC101905509, and LOC104972797. Moreover, their target protein-coding genes were MRPL14, ESPNL, GABPB1, KIAA0895L, and RIMS2, which were located in trans-position (Table 2).

**Table 2.** Prediction results of trans-target genes

mRNAs		mRNAs		
lncRNA_id	pos_cor_id	pos_cor	neg_cor_id	neg_cor
XR_808337.3	XM_024983717.1	0.992696064	XM_024994909.1	−0.998651441
XR_237807.3	XM_024983720.1	1	NM_001077128.2	−0.989386992
XR_001501504.2	XM_024983720.1	1	NM_001077128.2	−0.989386992
XR_003032689.1	XM_024983720.1	1	NM_001077128.2	−0.989386992
XR_003031215.1	XM_024983721.1	0.998164806	XM_010817975.3	−0.990428328
XR_003029770.1	XM_005204988.4	1	NM_001014887.1	−0.994701364
XR_003034054.1	XM_005204988.4	1	NM_001014887.1	−0.994701364
XR_003031220.1	XM_005204988.4	1	NM_001014887.1	−0.994701364
XR_003030419.1	XM_015467281.2	1	NM_001014887.1	−0.994701364
XR_001501114.2	XM_015467281.3	1	NM_001014887.1	−0.994701364
XR_001501115.2	XM_024997551.1	0.997605551	NM_001101148.2	−0.994808587
XR_003032366.1	XM_024997554.1	1	XM_002691498.5	−1
XR_236338.4	XM_024997554.1	1	XM_002691498.5	−1
XR_003037170.1	XM_024997554.1	1	XM_002691498.5	−1
XR_233741.4	XM_024997554.1	1	XM_002691498.5	−1
XR_001498511.2	XM_024978143.1	0.999999308	XM_005209141.4	−0.984748151
XR_003029635.1	XM_025001654.1	1	NM_001046128.1	−0.977564113
XR_807447.3	XM_025001664.1	0.999992755	XM_024983362.1	−0.985930072

lncRNA_id: ID of long non-coding RNA.

pos_cor_id: the co-expressed gene id of the largest positive correlation.

pos_cor: the coefficient of the largest positive correlation.

neg_cor_id: the co-expressed gene id with the largest negative correlation.

neg_cor: the coefficient of the largest negative correlation.

KEGG pathway enrichment analysis for DEGs could help to understand the signal transduction pathways that are activated or inhibited in host cells after BVDV infection and provide insights into the interactions between BVDV and its host. Our Illumina sequencing analysis of the alterations in gene expression of BVDV-stimulated MDBK cells provided important data on the specific aspects of molecular pathogenesis and virus–host interactions. Previous study has shown that BVDV strain NADL-infected MDBK cells were significantly enriched for target genes of lncRNAs that were involved in host immune responses, such as T cell receptor signaling pathway, TNF signaling pathway, and Jak-STAT signaling pathway [51], which were also found the enrichment of these target genes in the related pathways in our study. Moreover, in this study, we also found that many differentially expressed genes in cytopathic biotype BVDV-infected MDBK cells showed significant enrichment in NOD-like receptor signaling pathway, NF-κB signaling pathway, Hippo signaling pathway, and apoptosis pathway. All of these signaling pathways are involved in the regulation of MDBK cells stimulated by BVDV, providing useful data for the development of novel preventive or therapeutic strategies against BVDV. We also found that the significantly DE cis-and trans-acting lncRNAs contributed to regulation of autophagy signaling pathway, cytokine–cytokine receptor interaction, PI3K-Akt signaling pathway, and PPAR signaling pathway associated with the host immune response to BVDV infection. Undoubtedly, the transcriptome data obtained by RNA-seq will help us better understand the molecular mechanism of BVDV infection and host immune responses involved in host–virus interaction. Several DEGs detected in BVDV-infected MDBK cells were novel, alternative splicing isoforms, or related to signaling pathways. The expression of virus resistance genes could be part of the mechanisms by which BVDV escapes from the host immune defense system. Our data would provide a useful scientific reference for improving our understanding of the roles of host lncRNAs in the modulation of BVDV replication and pathogenesis.

In conclusion, we profiled the changes in expression of lncRNAs and mRNAs in BVDV-infected MDBK cells. GO and KEGG pathway enrichment analysis was used to identify all the DE mRNAs and lncRNAs. The analysis of BVDV-infected MDBK cells with comprehensive molecular data provided an insight into the roles of lncRNAs in BVDV infection and host immune responses involved in host–virus interaction. Moreover, further research is under way to investigate the functions of the DE lncRNAs to explore the mechanisms of BVDV infection and its pathogenesis.

## Supplementary Material

Supplemental Material
