# Supplementary material for: Comprehensive analysis of lncRNA expression profiles in cytopathic biotype BVDV-infected MDBK cells provides an insight into biological contexts of host–BVDV interactions
Source: Virulence. 2020 Dec 29;12(1):20–34. doi: 10.1080/21505594.2020.1857572 (PMC7781660; doi:10.1080/21505594.2020.1857572)
Supplement: Supplemental Material [file KVIR_A_1857572_SM0629.pdf]

Virulence

Supplementary files

Comprehensive analysis of lncRNA expression profiles in cytopathic biotype BVDV-infected MDBK cells provides an insight into biological contexts of host-BVDV interactions

Xuwen Gao[a,c]*, Chao Niu[a]*, Zhuo Wang[a], Shuo Jia[a], Meijing Han[a], Yingying Ma[a], Xueting Guan[a], Li Wang[a], Xinyuan Qiao[a], Yigang Xu[a,b]

[a.] Heilongjiang Key Laboratory for Animal Disease Control and Pharmaceutical Development, College of Veterinary Medicine, Northeast Agricultural University, Harbin, P.R. China
[b.] College of Animal Science and Technology & College of Veterinary Medicine, Zhejiang Agricultural and Forestry University, Hangzhou, P.R. China
[c.] College of Veterinary Medicine, China Agricultural University, Beijing, P.R. China

Figure legends

Figure S1. PCA analysis of differentially expressed mRNAs (**a**) and lncRNAs (**b**) in BVDV-infected MDBK cells. A two-dimensional coordinate graph is made according to the numerical value of each sample in two comprehensive indicators of the first principal component (PC1) and the second principal component (PC2). The number in parentheses on the axis labels represents the percentage of the principal component explaining the overall variance.

Figure S2. Correlation heat map. According to the quantitative results of FPKM, the correlation between each sample was calculated, and the correlation heat map was drawn using R language to test the reliability of the experiment and the rationality of sample selection. **a**) Heat map of mRNA correlation. **b**) Heat map of lncRNA correlation.

Figure S3. Heat map of differentially expressed mRNAs in BVDV-infected MDBK cells (blue represents mock-infected MDBKs; red represents BVDV-infected MDBKs). C1, C2, and C3 are the mock-infected MDBK groups. E1, E2, and E3 are the BVDV-infected MDBK groups. The transcript expression of each sample was calculated by a log2, and hierarchical analysis was performed on different samples and transcripts. Each column represents one sample and each row represents one transcript. The transcripts are from different samples. The level of expression is represented by different colors. The redder the color, the higher the expression level, while the bluer the color, the lower the expression level.

Figure S4. Heat map of differentially expressed lncRNAs in BVDV-infected MDBK cells (blue represents mock-infected MDBKs; red represents BVDV-infected MDBKs). C1, C2, and C3 are the mock-infected MDBK groups. E1, E2, and E3 are the BVDV-infected MDBK groups. The transcript expression of each sample was calculated by a log2, and hierarchical analysis was performed on different samples and transcripts. Each column represents one sample and each row represents one transcript. The transcripts are from different samples. The level of expression is represented by different colors. The redder the color, the higher the expression level, while the bluer the color, the lower the expression level.

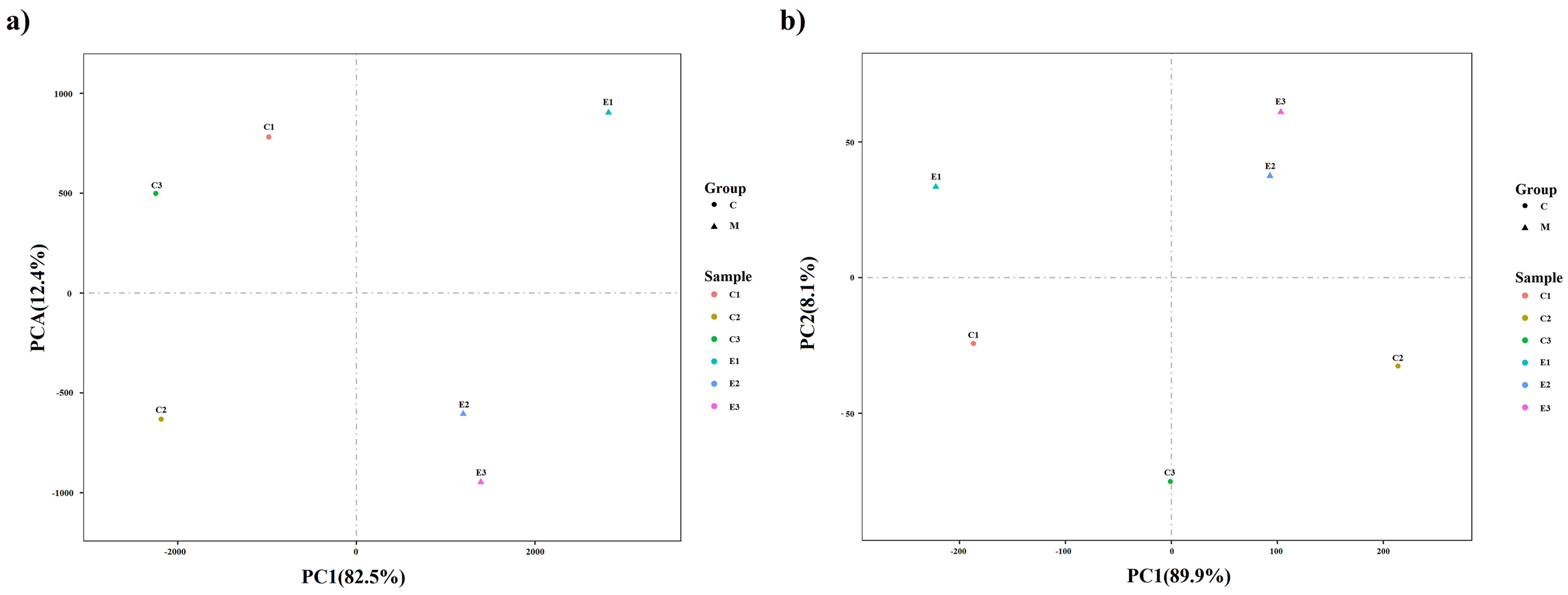
a)
PCA(12.4%)
PC1(82.5%)
1000
500
0
-500
-1000
-2000
0
2000
C1
C2
C3
E1
E2
E3
Group
C
M
Sample
C1
C2
C3
E1
E2
E3
b)
PC2(8.1%)
PC1(89.9%)
50
0
-50
-200
-100
0
100
200
C1
C2
C3
E1
E2
E3
Group
C
M
Sample
C1
C2
C3
E1
E2
E3

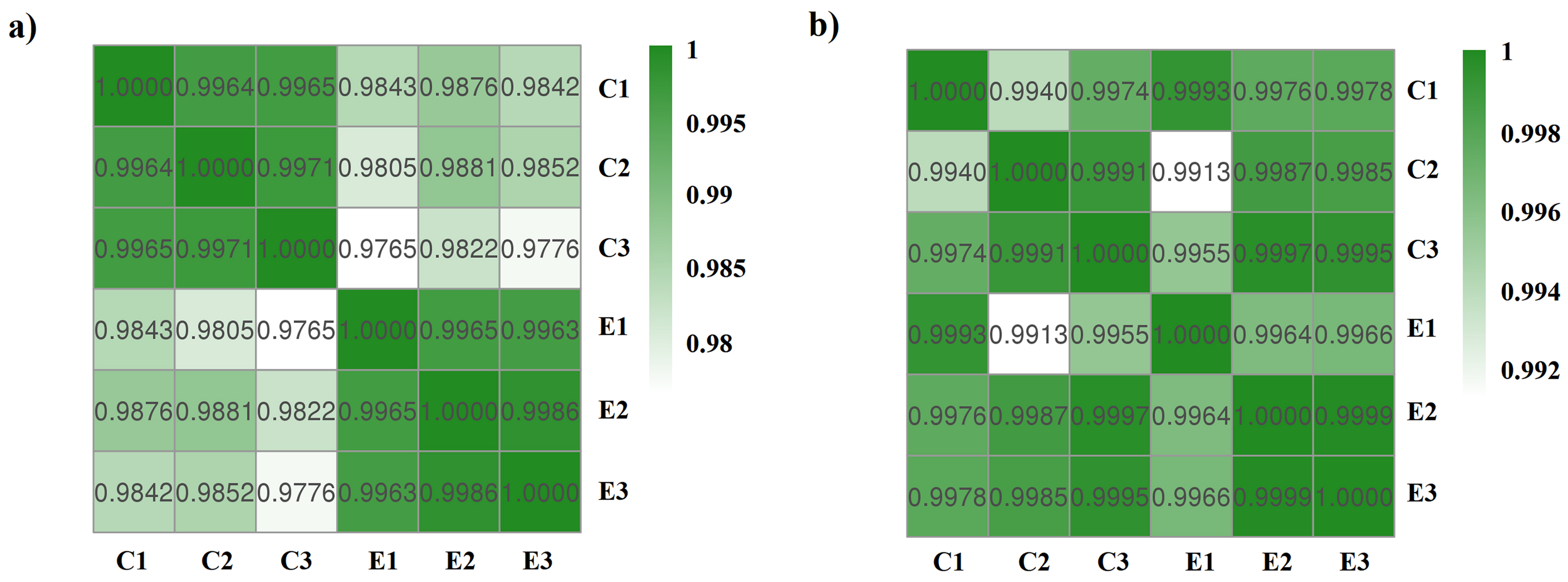
a)
1.0000 0.9964 0.9965 0.9843 0.9876 0.9842 C1
0.9964 1.0000 0.9971 0.9805 0.9881 0.9852 C2
0.9965 0.9971 1.0000 0.9765 0.9822 0.9776 C3
0.9843 0.9805 0.9765 1.0000 0.9965 0.9963 E1
0.9876 0.9881 0.9822 0.9965 1.0000 0.9986 E2
0.9842 0.9852 0.9776 0.9963 0.9986 1.0000 E3
C1 C2 C3 E1 E2 E3
1
0.995
0.99
0.985
0.98
b)
1.0000 0.9940 0.9974 0.9993 0.9976 0.9978 C1
0.9940 1.0000 0.9991 0.9913 0.9987 0.9985 C2
0.9974 0.9991 1.0000 0.9955 0.9997 0.9995 C3
0.9993 0.9913 0.9955 1.0000 0.9964 0.9966 E1
0.9976 0.9987 0.9997 0.9964 1.0000 0.9999 E2
0.9978 0.9985 0.9995 0.9966 0.9999 1.0000 E3
C1 C2 C3 E1 E2 E3
1
0.998
0.996
0.994
0.992

Figure S3

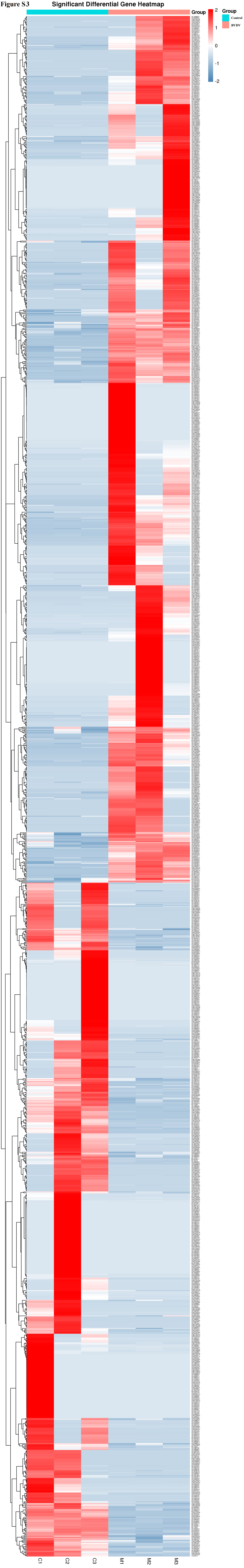
Significant Differential Gene Heatmap
Group
Group
Control
BVDV
2
1
0
-1
-2
C1
C2
C3
M1
M2
M3

Figure S4

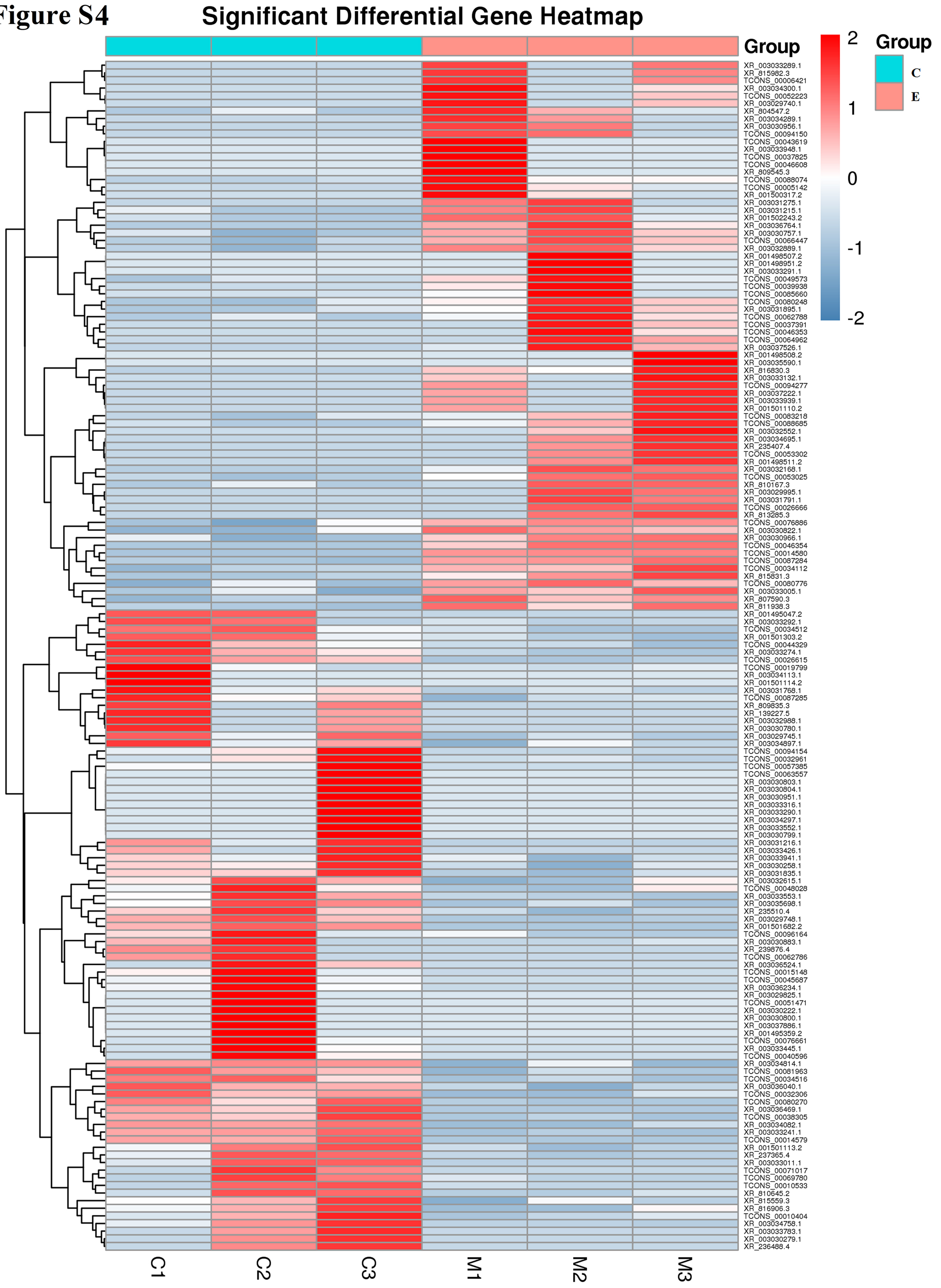
Significant Differential Gene Heatmap
Group
2
1
0
-1
-2
Group
C
E
C1
C2
C3
M1
M2
M3
